# The Birth of the Contextual Health Education Readability Score in an Examination of Online Influenza Patient Education Materials

**DOI:** 10.7759/cureus.56715

**Published:** 2024-03-22

**Authors:** Bilal Irfan, Ihsaan Yasin, Aneela Yaqoob

**Affiliations:** 1 Microbiology and Immunology, University of Michigan, Ann Arbor, USA; 2 Electrical Engineering and Computer Science, University of Michigan, Ann Arbor, USA; 3 Infectious Diseases, Beaumont Hospital, Taylor, USA

**Keywords:** online health resources, actionability, contextual health education, health literacy, readability assessment, patient education materials, influenza education

## Abstract

Introduction

Influenza is a major global health concern, with its rapid spread and mutation rate posing significant challenges in public health education and communication. Effective patient education materials (PEMs) are crucial for informed decision-making and improved health outcomes. This study evaluates the efficacy of online influenza PEMs using traditional readability tools and introduces the Contextual Health Education Readability Score (CHERS) to address the limitations of existing methods that do not capture the diverse array of visual and thematic means displayed.

Materials and methods

A comprehensive search was conducted to select relevant online influenza PEMs. This involved looking through Google's first two pages of results sorted by relevance, for a total of 20 results. These materials were evaluated using established readability tools (e.g., Flesch Reading Ease, Flesch-Kincaid Grade Level) and the Patient Education Materials Assessment Tool (PEMAT) for understandability and actionability. The study also involved the creation of CHERS, integrating factors such as semantic complexity, cultural relevance, and visual aid effectiveness. The development of CHERS included weighting each component based on its impact on readability and comprehension.

Results

The traditional readability tools demonstrated significant variability in the readability of the selected materials. The PEMAT analysis revealed general trends toward clarity in purpose and use of everyday language but indicated a need for improvement in summaries and visual aids. The CHERS formula was calculated as follows: CHERS = (0.4 × Average Sentence Length) + (0.3 × Average Syllables per Word) + (0.15 × Semantic Complexity Score) + (0.1 × Cultural Relevance Score) + (0.05 × Visual Aid Effectiveness Score), integrating multiple dimensions beyond traditional readability metrics.

Discussion

The study highlighted the limitations of traditional readability tools in assessing the complexity and cultural relevance of health information. The introduction of CHERS addressed these gaps by incorporating additional dimensions crucial for understanding in a healthcare context. The recommendations provided for creating effective influenza PEMs focused on language simplicity, cultural sensitivity, and actionability. This may enable further research into evaluating current PEMs and clarifying means of creating more effective content in the future.

Conclusions

The study underscores the need for comprehensive readability assessments in PEMs. The creation of CHERS marks a significant advancement in this field, providing a more holistic approach to evaluating health literacy materials. Its application could lead to the development of more inclusive and effective educational content, thereby improving public health outcomes and reducing the global burden of influenza. Future research should focus on further validating CHERS and exploring its applicability to other health conditions.

## Introduction

​​Influenza, or the flu, stands as a significant public health challenge, affecting populations worldwide with its highly contagious nature [1]. Characterized by symptoms such as fever, cough, and malaise, influenza causes considerable morbidity and mortality annually [2]. With approximately a billion cases each year, including 3-5 million severe illnesses and 290,000 to 650,000 respiratory deaths globally per the World Health Organization (WHO), the flu’s impact is profound and far-reaching [3]. Its rapid transmission in crowded areas, such as schools and nursing homes, exacerbates its spread, making it a formidable foe in public health.

The difficulties in tackling influenza are manifold. Firstly, its high mutation rate enables it to evade the immune response, leading to annual outbreaks [4]. This necessitates constant vigilance and updates in vaccine composition, as seen in the recommendations for the 2023-2024 influenza season by the American Academy of Pediatrics [5]. Given this context, it is crucial to understand that while influenza poses a threat to the general population, its impact is disproportionately severe among high-risk groups. This includes pregnant women, children under five, older adults, and individuals with chronic medical conditions, underscoring the need for targeted education and prevention strategies [6]. Educational materials and campaigns that focus on the critical role of influenza vaccination in safeguarding susceptible populations, along with enhancing awareness and countering misinformation, can be profoundly effective [7].

However, educating populations about influenza is fraught with challenges, particularly in developing countries where resources and access to health information are often limited. These barriers include a lack of awareness, misconceptions about the disease and its vaccination, and varying levels of health literacy among the population [8]. Moreover, logistical issues such as the distribution of materials, language barriers, and cultural differences can further complicate the effective dissemination of health information. Additionally, the presence of conflicting information from unofficial sources can undermine trust in official health advisories. In such scenarios, patient education materials (PEMs) emerge as vital tools in healthcare communication. PEMs are designed to inform and guide patients about various aspects of health and diseases, including their causes, prevention, management, and treatment options [9]. They play a crucial role in empowering patients with knowledge, thereby aiding in informed decision-making and improved health outcomes.

Yet, the effectiveness of PEMs is contingent upon their readability, understandability, and actionability. These attributes determine how well the target audience can comprehend and use the information provided. Readability refers to the ease with which a reader can understand the written text, while understandability encompasses how well the material conveys its message. Actionability indicates the extent to which the reader can use the information to make informed health decisions. To assess these aspects, various readability tools are employed. For example, in a scenario where PEMs are used to explain the importance of flu vaccination, high readability and understandability can lead to better comprehension among elderly patients, thereby increasing their likelihood of getting vaccinated and reducing the incidence of flu-related complications in this high-risk group. These tools, such as the Flesch Reading Ease, Flesch-Kincaid Grade Level, and others, provide quantitative measures of the text's complexity based on factors like sentence length and word difficulty [10]. However, these tools have their limitations as they do not account for the content's contextual or cultural relevance. In addition to readability tools, the Patient Education Materials Assessment Tool (PEMAT) is another crucial instrument used in evaluating health information. PEMAT allows for a more comprehensive assessment of both understandability and actionability. It involves a systematic evaluation of the material's presentation, content, and design to ensure it meets the needs of diverse patient populations [11].

This study aims to evaluate current online influenza PEMs using a range of readability tools and PEMAT to determine their effectiveness in communicating crucial health information to the public. By analyzing these materials, the study seeks to identify areas for improvement and contribute to the development of more effective patient education strategies for influenza prevention and management. Such efforts are essential in reducing the burden of influenza, especially among vulnerable populations, and enhancing public health outcomes. In that endeavor, a novel evaluation tool has been developed, the Contextual Health Education Readability Score (CHERS). It is presented to address the current gaps by quantitatively evaluating PEMs by integrating factors such as semantic complexity, cultural relevance, and visual aid effectiveness, along with traditional readability measures.

## Materials and methods

The methodology of this study was designed to systematically evaluate the quality of online PEMs related to influenza. On January 2, 2024, a comprehensive search was conducted using Google, given its widespread popularity, with the search term "flu patient education." This date was chosen to ensure the most current information was available following the annual updates in the flu vaccine and treatment guidelines, which are typically released prior to the flu season each year.

The search resulted in a list of articles from various sources. The first two pages of Google search results were screened, yielding a total of 20 articles. These articles were then subjected to a set of inclusion criteria that rested on determining their relevance as PEMs. Attention was paid to ensure that the intended audience was patients or an average person seeking to undertake preventive measures or appropriate treatment, rather than advice for physicians or healthcare providers. The criteria included factors such as the material being publicly accessible, specifically focused on influenza, and utilizing terminology and verbiage that clearly indicates it is intended for a general audience. After applying these criteria, 18 articles were selected for detailed evaluation by the authors.

Each selected article underwent a readability assessment using a suite of established readability tools. These tools included the Flesch Reading Ease, Flesch-Kincaid Grade Level, Gunning Fog Index, SMOG (Simple Measure of Gobbledygook) Index, Automated Readability Index, Spache Formula, Dale-Chall Formula, Powers-Sumner-Kearl, Raygor Readability Graph, Coleman Liau Index, Fry Readability Graph, IELTS (International English Language Testing System), CEFR (Common European Framework of Reference for Languages), Lix and Rix Readability Formulas, Lensear Write Readability Formula, and FORCAST Readability Formula. These tools provide quantitative data on the complexity of the text based on syntax and word choice, thus offering a multifaceted view of the material's readability [12].

In addition to readability scores, each article was evaluated using the PEMAT for understandability and actionability. This assessment was performed by two independent reviewers. Scores assigned by the reviewers were averaged to obtain a final PEMAT score for each article, ensuring a balanced and unbiased evaluation. To ensure the reliability of the assessments, inter-rater reliability was calculated for the PEMAT scores. This involved comparing the scores assigned by the two reviewers and measuring the level of agreement between them, which was found to be satisfactory. High inter-rater reliability indicates consistency in the evaluation process, which is crucial for the validity of the study's findings [13]. 

The development of recommendations for creating effective influenza PEMs was based on a detailed analysis of the preliminary findings noted above, and a thorough review of best practices in health communication and patient education literature. This entailed examining the key elements that influence the effectiveness of educational materials, including language simplicity, cultural sensitivity, and the use of visual aids [14]. Recognizing the diversity of the target audience, the recommendations were designed to address various aspects of inclusivity and comprehension. The team consulted with experts in public health, health literacy, and cultural communication from across disciplines and institutions who offered feedback or insights to ensure the recommendations were practical and grounded in current best practices. This collaborative approach ensured the recommendations were well-rounded, addressing the needs of both healthcare providers creating the materials and the diverse patient populations consuming them. In addition to this, sample survey prompts were created for future studies to potentially utilize. They were designed to capture both qualitative and quantitative elements, allowing for a rich analysis of public perceptions and experiences. Two independent reviewers were tasked with ensuring questions were clear, concise, and unbiased to facilitate ease of response and accuracy in data collection for any future usage.

For the development of the Contextual Health Education Readability Score (CHERS), a systematic methodology was adopted. After identifying the key components for the tool, the next step involved determining the appropriate weight for each component. This decision was based on the relative importance of each factor in enhancing the readability and effectiveness of PEMs. The team engaged in an iterative process, examining a range of existing materials and applying different weight combinations to assess their impact on the final readability score. Pilot testing of the CHERS tool was crucial in its development, which was conducted through a selection of five influenza PEMs that were scored using the CHERS formula. The rough results were then compared with the responses of the other tests and feedback from independent reviewers. Adjustments were made consequently to the scoring weight, ensuring that the CHERS tool accurately reflected the readability and comprehensibility of the materials from the perspective of a potential intended audience.

## Results

The results of the existing tests revealed significant variations in the readability of influenza patient education materials. The readability assessment demonstrated a range of scores across different sources (Table 1). 

**Table 1:** Readability assessment tests for influenza patient education materials SMOG: Simple Measure of Gobbledygook; IELTS: International English Language Testing System; CEFR: Common European Framework of Reference for Languages; UCSF: University of California San Francisco; CDC: Centers for Disease Control and Prevention; AOA: American Osteopathic Association

Source	Flesch Reading Ease	Flesch Kincaid Grade Level	Gunning Fog Index	SMOG Index	Automated Readability Index	Spache Formula	Dale-Chall Formula	Powers-Sumner-Kearl	Raygor Readability Graph	Cole-man Liau Index	Fry Readability Graph	IELTS	CEFR	Lix and Rix Readability Formulas	Lensear Write Readability Formula	FORCAST Readability Formula
UpToDate (Symptoms and Treatment)	51.25	11.53	14.72	13.19	13.08	5.5	9.5	8.5	10.5	11.5	10.5	7.0	B2/C1	55, 6.5	11	11.5
UCSF	57.48	9.61	12.91	12.31	10.47	4.8	9.7	8.4	10.6	11.36	9.8	6.8	B2.2	50.3, 5.8	9.6	10.7
UpToDate (Prevention)	50.5	9.72	13.32	12.53	11.06	5.2	10.1	8.6	10.8	13.85	10.2	7.0	B2.3	52.1, 6.1	10	11
CDC	78.98	5.585	8.56	8.84	6.07	4.9	5.9	6.2	6.4	7.43	5.7	5.5	B1	35.2, 4.1	5.8	6.0
AOA	42.6	12.35	15.65	14.3	13.1	5.4	10.2	8.7	11	13.21	10.4	7.1	B2.4	53.2, 6.2	10.1	11.2
My Health Alberta	73.91	6.01	8.4	9.04	6.78	4.6	5.8	6.5	7.2	9.33	6.7	5.6	B1.1	40.5, 4.6	6.2	7.0
American Thoracic Society	61.81	8.33	11.56	11.38	9.05	5.3	6.1	7	8.5	10.97	7.8	6.2	B2	46.3, 5	7.5	8.3
KidsHealth	72.4	6.7	8.6	9.2	7.3	4.6	5.9	6.2	7.8	10.4	6.4	5.7	B1.3	38,7, 4.4	6.5	7.1
CDC (Prevention)	70.3	6.8	8.5	8.9	7.2	4.7	6.3	6.9	7.4	9.6	7.1	5.8	B1.4	41.2, 4.5	6.9	7.3
Families Fighting Flu	68.7	6.5	8.9	9.3	7.4	4.8	6.4	7.1	7.5	10.6	7.2	6.0	B1.5	42.3, 4.6	7.0	7.4
UC Davis Health	64.8	7.6	9.7	10.2	8.3	5.2	6.7	7.4	8.1	11.1	7.9	6.1	B2.1	43.6, 4.9	7.8	8.2
Elsevier	63.2	8.1	10.4	10.7	8.6	5.6	7.1	7.9	8.5	11.3	8.2	6.3	B2.2	45.1, 5.2	8.0	8.8
U.S. Pharmacist	61.7	8.9	11.3	11.6	9.2	5.9	7.8	8.4	9.0	12.5	8.7	6.5	B2.4	47.8, 5.7	9.1	9.4
Mayo Clinic	62.9	8.3	10.8	11.1	8.9	5.7	7.3	8.0	8.7	11.6	8.4	6.4	B2.3	46.8, 5.3	8.2	9.1
UC Davis Health (Parents of Children)	62.4	8.3	10.5	10.8	8.7	5.7	7.2	8.0	8.6	11.4	8.3	6.4	B2.3	46.2, 5.3	8.1	8.9
UCSF Health (Staying Healthy)	79.3	5.6	7.2	7.8	6.3	4.4	5.9	6.5	6.8	8.9	6.3	5.6	B1.2	38.7, 3.9	5.8	6.2
Allegheny Health Network	65.2	7.9	9.6	9.9	8.4	5.3	6.8	7.7	8.2	10.7	7.9	6.2	B2.1	44.9, 5.0	7.6	8.5
UCSF: Benioff Children’s Hospitals	63.7	8.2	10.6	10.9	8.8	5.8	7.3	8.1	8.7	11.5	8.4	6.5	B2.4	47.0, 5.4	8.2	9.0

For instance, the Flesch Reading Ease scores varied from 42.6 (AOA) to 79.3 (UCSF Health - Staying Healthy), indicating differences in the complexity of the text. Similarly, the Flesch Kincaid Grade Level scores ranged from 5.585 (CDC) to 12.35 (AOA), suggesting variability in the grade level of the materials. The Gunning Fog Index, SMOG Index, and other readability tools reflected similar disparities. These variations highlight the diverse nature of the readability of online PEMs on influenza.

The PEMAT results, which evaluated the understandability and actionability of the materials, revealed certain broad consistencies in general trends (Table 2). 

**Table 2:** PEMAT results for influenza patient education materials PEMAT: Patient Education Materials Assessment Tool; PEMAT-P: Patient Education Materials Assessment Tool for Printable Materials

Understandability and Actionability (PEMAT-P) Scoring Criteria	Number of articles (n=18)
The material makes its purpose completely evident.	18
The material does not include information or content that distracts from its purpose.	17
The material uses common, everyday language.	17
Medical terms are used only to familiarize audience with the terms. When used, medical terms are defined.	16
The material uses the active voice.	16
Numbers appearing in the material are clear and easy to understand. (No numbers = N/A)	13/17
The material does not expect the user to perform calculations.	18
The material breaks or "chunks" information into short sections. (Very short material = N/A)	17/17
The material's sections have informative headers. (Very short material = N/A)	17/17
The material presents information in a logical sequence.	18
The material provides a summary. (Very short material = N/A)	2/17
The material uses visual cues (e.g., arrows, boxes, bullets, bold, larger font, highlighting) to draw attention to key points.	13
The material uses visual aids whenever they could make content more easily understood (e.g., illustration of healthy portion size).	8
The material’s visual aids reinforce rather than distract from the content. (No visual aids = N/A)	6/8
The material’s visual aids have clear titles or captions. (No visual aids = N/A)	4/8
The material uses illustrations and photographs that are clear and uncluttered. (No visual aids = N/A)	8/8
The material uses simple tables with short and clear row and column headings. (No tables = N/A)	N/A
The material clearly identifies at least one action the user can take.	14
The material addresses the user directly when describing actions.	13
The material breaks down any action into manageable, explicit steps.	14
The material provides a tangible tool (e.g., menu planners, checklists) whenever it could help the user take action.	2
The material provides simple instructions or examples of how to perform calculations. (No calculations = N/A)	N/A
The material explains how to use the charts, graphs, tables, or diagrams to take actions. (No charts, graphs, tables, or diagrams = N/A)	N/A
The material uses visual aids whenever they could make it easier to act on the instructions	3

The study found that all 18 evaluated materials made their purpose completely evident and presented information in a logical sequence. However, only a minority of the materials provided a summary (2/17) and used visual aids to make it easier to act on the instructions (3). The PEMAT scores indicated that while most materials were clear in their purpose and used everyday language (17/18), there was room for improvement in using visual aids and providing actionable content. These findings suggest that while the materials are generally understandable, their ability to drive action could be enhanced (Table 3).

**Table 3:** Recommendations for creating effective influenza patient education materials

Category	Recommendation	Rationale/Details
Language Use	Simplify medical jargon	Avoid medical jargon and use layman's terms for better comprehension. E.g., use “flu” instead of “influenza”. When technical terms are necessary, include simple definitions.
	Translate materials into multiple languages.	Accommodate linguistic diversity; offer translations to cater to non-English speaking audiences.
	Be concise and clear	Use short sentences and paragraphs. Avoid complex syntax or overly long explanations.
Design Principles	Use visual aids	Include charts, diagrams, and illustrations to explain concepts like the spread of influenza or preventive measures. Ensure visuals are clear and directly related to the text.
	Layout and Typography	Improve readability and focus on key information using easy-to-read fonts and avoiding dense text blocks. Use headings and bullet points for easy scanning, and a clean layout with legible fonts and sufficient white space.
Cultural Considerations	Cultural Relevance and Sensitivity	Address diverse health perceptions and practices to increase material relevance and effectiveness. E.g., medications to be Kosher or Halal-compliant. Include scenarios or examples that resonate with different communities.
	Use culturally diverse images.	Reflect audience diversity in visuals for inclusiveness and better engagement. E.g., taking oral medications with a niqab.
Actionability	Clearly identify actionable steps.	Provide specific, clear instructions for actions like vaccination or hygiene practices.
	Break down complex actions into manageable steps.	Simplify complex recommendations into step-by-step instructions for easier adherence.
Engagement Strategies	Include interactive elements (e.g., quizzes, checklists).	Enhance engagement and retention of information through interactive content.
Accessibility	Ensure materials are accessible to people with disabilities.	Include alternative text for visuals, accessible web design, and consider readability for various disabilities.
Feedback Mechanism	Provide a way for readers to give feedback.	Use feedback to improve future materials and address specific community needs or concerns.

CHERS is a novel readability formula designed to offer a more comprehensive evaluation of PEMs, particularly in the health context. It moves beyond traditional readability measures, factoring in the complexity of medical information and the cultural and visual aspects of patient education. This multidimensional approach aims to ensure that the materials are not only linguistically accessible but also contextually relevant and engaging for diverse audiences. The inclusion of semantic complexity and cultural relevance in CHERS is particularly crucial in health education, where understanding complex medical terms and cultural sensitivity can significantly impact patient comprehension and adherence to health advice. By incorporating these elements, CHERS provides a more nuanced assessment of the materials, aiding healthcare providers and educators in developing more effective and inclusive patient education resources. The calculation of CHERS for each material involved five key components: Average Sentence Length, Average Syllables per Word, Semantic Complexity Score, Cultural Relevance Score, and Visual Aid Effectiveness Score. Each component was assigned a specific weight based on its perceived impact on readability and comprehension in a healthcare context (Table 4). 

**Table 4:** Calculation method for CHERS CHERS: Contextual Health Education Readability Score

Component	Weight in CHERS Formula	Description	Scoring/Measurement Method
Average Sentence Length	0.4	The average number of words in a sentence.	Count the total number of words and divide by the number of sentences to get the average sentence length.
Average Syllables per Word	0.3	Average complexity of words based on syllable count.	Calculate the average number of syllables across a representative sample of words from the material.
Semantic Complexity Score	0.15	The complexity of medical concepts and terminology used.	Rate on a scale of 1-5 (1 being basic, 5 being highly complex). This involves assessing how advanced or specialized the medical concepts and terms used in the material are.
Cultural Relevance Score	0.1	The degree to which the material is culturally sensitive and relevant.	Rate on a scale of 1-5 (1 being not culturally relevant, 5 being highly culturally relevant). This is based on how well the material considers diverse cultural backgrounds.
Visual Aid Effectiveness Score	0.05	The effectiveness of visual aids in supporting text comprehension.	Rate on a scale of 1-5 (1 being ineffective, 5 being highly effective). Evaluate the clarity, relevance, and integration of visual aids in enhancing understanding of the text.

The survey prompts created were designed to help future researchers or studies gauge public perception and understanding of influenza PEMs. These questions cover various aspects, such as the frequency of seeking flu information online, ease of understanding the materials, and the effectiveness of provided visual aids and actionable steps. These prompts can also be specifically tailored for other conditions or viruses. The goal of these prompts is to assess how well these materials meet the needs of the general public and to identify areas for improvement in future patient education strategies (Table 5).

**Table 5:** Sample prompts for a potential survey on influenza patient education materials

Question	Response Options
How often do you seek information about influenza (the flu) online?	Never Rarely Sometimes Often Always
Where do you usually find information about influenza? (Select all that apply)	Health websites (e.g., WebMD, Mayo Clinic) Government health sites (e.g., CDC, WHO) Social media (e.g., Instagram, Facebook, X) Printed materials (e.g., brochures, flyers) Healthcare providers Other (Please specify): __________
How easy is it for you to understand the information provided in online influenza patient education materials?	Very difficult Somewhat difficult Neutral Somewhat easy Very easy
Do you find that the language used in these materials is clear and easy to understand?	Strongly disagree Disagree Neutral Agree Strongly agree
How often do these materials provide actionable steps for flu prevention or treatment?	Never Rarely Sometimes Often Always
Do you feel confident in your ability to follow the advice or instructions provided in these materials?	Not confident at all Slightly confident Moderately confident Very confident Extremely confident
How often do the materials include visual aids (like pictures or diagrams) to help explain the information?	Never Rarely Sometimes Often Always
Do you think these materials are culturally sensitive and relevant to diverse audiences?	Not at all Slightly Moderately Mostly Completely
After reading these materials, how likely are you to take preventive measures against influenza (like getting vaccinated)?	Very unlikely Unlikely Neutral Likely Very likely
Would you recommend these educational materials to others seeking information about influenza?	Definitely not Probably not Might or might not Probably would Definitely would

## Discussion

The analysis of influenza PEMs through various readability tools and PEMAT has yielded critical insights into the current state of online flu education resources. The significant variability in readability scores across the selected materials underscores the diverse nature of these resources. This variability is crucial because it reflects the potential challenges in ensuring that the materials are accessible and understandable to a broad audience. For instance, higher grade level scores in some materials suggest they might be less accessible to individuals with lower literacy levels. This discrepancy in readability levels raises concerns about the equitable dissemination of vital health information, especially considering the widespread impact of influenza and the need for widespread public understanding of prevention and treatment strategies.

The PEMAT results indicate a general trend toward clarity in purpose and use of everyday language, which is commendable. However, the limited use of summaries and visual aids to facilitate actionability points to a significant area for improvement. This is particularly important in health communication, where the ultimate goal is not just to inform but also to enable individuals to make informed health decisions and take appropriate actions, such as seeking vaccination or recognizing flu symptoms early [15]. The inclusion of visual aids could help facilitate easier comprehensibility of actions for people, particularly in certain treatments or preventive measures such as hand washing where step-by-step guidance that can be easily replicated can be an asset.

The inclusion of survey prompts in this study represents a pivotal step toward understanding public engagement with influenza PEMs. The survey, designed with a focus on clarity and comprehensiveness, aims to capture the real-world experiences and perceptions of individuals seeking flu-related information. By exploring aspects such as the frequency of information seeking, ease of understanding, and the perceived effectiveness of visual aids, the gap between theoretical readability scores and actual user experiences can be bridged. This approach is critical in identifying practical shortcomings and strengths of current materials, thereby guiding the development of more user-centric educational content. The survey prompts with their emphasis on actionable steps and cultural sensitivity serve as a vital tool for future researchers, enabling a more nuanced understanding of public needs in health literacy. It could also potentially serve to aid in the creation of a new tool or refinement of the existing one.

The recommendations outlined above are the cornerstone of the initiative to enhance the effectiveness of influenza PEMs. They encompass a range of strategies, from simplifying medical jargon to using culturally sensitive imagery, aimed at making health information more accessible and relatable. The focus on language use and design principles underscores the importance of clear communication in medical contexts. By advocating for concise language, multilingual translations, and the strategic use of visual aids, these recommendations address key barriers to health literacy. Furthermore, the emphasis on cultural considerations and actionability in our recommendations reflects a deep understanding of the diverse backgrounds and practical needs of the audience. These guidelines serve as a blueprint for healthcare providers to create materials that are not only informative but also empowering, ensuring that crucial health messages are received and understood by all segments of the population.

The diverse range of readability scores from the various tools also reflects the complex nature of health literacy. Health literacy is not just about reading ability; it encompasses a range of skills, including numeracy, comprehension, and the ability to apply information to health-related decisions [16]. The high readability scores of some materials could impede understanding among populations with varying levels of health literacy, which is a significant public health concern. Health information must be accessible not only in terms of language but also in format and presentation [17]. The use of technical or medical jargon, even when well-explained, can be a barrier. While some materials successfully defined medical terms, the sheer volume of technical information might still overwhelm or alienate some readers. This emphasizes the need for a balance between scientific accuracy and approachability in PEMs.

The analysis also sheds light on the crucial role of visual aids in enhancing comprehension and recall. Visual aids are not just supplementary but can be central to understanding complex health information [18]. The limited use of effective visuals in the evaluated materials is a missed opportunity, particularly in an era where digital media consumption often involves visual engagement. The integration of infographics, videos, and interactive elements could significantly enhance the actionability of these materials. Furthermore, the results highlight a potential mismatch between the content of these materials and the real-life context of the patients. Understanding the patient's perspective, cultural background, and health beliefs is essential in designing effective PEMs. Influenza PEMs should include consideration of psychological variables in regard to the intent to receive influenza vaccination, with communication of vaccine safety and emphasis on the potential risks of the disease [7]. This aspect of contextuality is not captured by readability scores and PEMAT alone, suggesting a need for more holistic approaches to evaluating PEMs.

The development and implementation of the CHERS represents a significant advancement in the evaluation of PEMs. Unlike traditional readability formulas, CHERS incorporates critical aspects such as semantic complexity, cultural relevance, and visual aid effectiveness, offering a more holistic assessment of the materials. This multidimensional approach is crucial in addressing the nuanced needs of diverse audiences, ensuring that health information is not only easy to read but also contextually appropriate and engaging. The adaptability of CHERS across different health topics and demographic groups makes it a versatile tool for future research and practice in health communication. Its application could lead to the creation of more inclusive and effective health education materials, ultimately contributing to better health outcomes. This can especially be considered in the context of the fact that the field of infectious diseases has had diminished interest with respect to other medical specialties, and there is a burgeoning need to consider the role of information dissemination in promoting healthcare equity, culturally conscious care, and general public health [19-23]. The potential of CHERS in transforming health literacy practices is immense, particularly in an era where accurate and accessible health information is more crucial.

Limitations

This study, while insightful, is not without limitations. Firstly, the reliance on Google search results may introduce a selection bias as the ranking of articles could be influenced by factors beyond their quality or relevance, such as search engine optimization strategies [24]. Looking at Internet Explorer, Bing, and other commonly used browsers as well as pooling for a larger sample size by looking at perhaps the first hundred or so results would allow researchers to paint a broader picture of PEMs. Secondly, the readability tools used, despite their widespread acceptance, have inherent limitations. They primarily focus on linguistic and syntactic elements of the text and do not account for factors such as cultural relevance, context, or the use of visual aids, which can significantly impact a reader's understanding. While a new tool was created as a result, its implementation and validation can continue to be strengthened through usage by other researchers. Additionally, the PEMAT, while comprehensive, is subject to the subjective interpretations of the reviewers, and different reviewers might score the same material differently.

Furthermore, the study's focus on online materials excludes a segment of the population without internet access, which is particularly relevant in lower-income and rural areas. Future studies could aim to incorporate offline materials or strategies to reach these populations. This highlights a digital divide in health information access, which can perpetuate health inequalities [25]. Lastly, the cross-sectional nature of this study limits its ability to track changes over time, especially in response to emerging flu strains or new public health guidelines. Other tools have also been developed across other disciplines for analyzing audiovisual content, which could serve as a sounding board for the refinement of existing measures [26,27]. 

## Conclusions

The study underscores the importance of developing PEMs that are not only scientifically accurate but also tailored to meet the varied needs of the general public. This involves considering factors such as cultural sensitivity, language simplicity, and the effective use of visuals. As influenza continues to pose a significant public health threat, the role of PEMs in educating and empowering the public becomes ever more critical. Future efforts should focus on addressing the identified gaps and continually adapting materials to align with emerging health literacy needs and technological advancements. This can include incorporating real-time updates and other interactive elements into the materials produced. By doing so, healthcare providers and public health authorities can better equip individuals to understand and respond effectively to the challenges posed by influenza, ultimately contributing to improved health outcomes and a reduction in the global burden of this pervasive disease.

The creation and integration of CHERS into our study marks a transformative step in evaluating and enhancing PEMs. This innovative tool transcends traditional readability assessments by incorporating critical factors like semantic complexity, cultural relevance, and the effectiveness of visual aids. CHERS stands as a testament to the evolving needs of health literacy, offering a more comprehensive and nuanced understanding of PEMs' effectiveness. Its application promises to revolutionize the way healthcare providers develop and distribute information, ensuring that it resonates with and is accessible to diverse audiences. As we continue to navigate the challenges posed by influenza and other public health issues, tools like CHERS will play an instrumental role in empowering individuals with the knowledge needed to make informed health decisions. The adoption of CHERS could significantly impact the future of patient education, making health information not just more readable but genuinely comprehensible and actionable across various cultural and linguistic landscapes.
